# Data on Drosophila clots and hemocyte morphologies using GFP-tagged secretory proteins: Prophenoloxidase and transglutaminase

**DOI:** 10.1016/j.dib.2019.104229

**Published:** 2019-07-09

**Authors:** Alexis Dziedziech, Martin Schmid, Badrul Arefin, Thomas Kienzle, Robert Krautz, Ulrich Theopold

**Affiliations:** Stockholm University, Department of Molecular Biosciences, Svante Arrhenius Väg 20C, SE, 10691 Sweden

**Keywords:** Insect immunity, Innate immunity, Non-classical secretion, Transglutaminase, Prophenoloxidase, Coagulation, Hemocytes

## Abstract

Insect hemolymph coagulation: Kinetics of classically and non-classically secreted clotting factors Schmid et al., 2019. The linked article demonstrates the localization of two secretory proteins in *Drosophila melanogaster*, Prophenoloxidase (PPO2) and Transglutaminase-A (Tg) in hemocytes as well the clot with different tissue-specific drivers. Here we provide further data for the usefulness of the GFP-tagged version of the two crosslinking enzymes that are involved in clot hardening. The morphology of crystal cells is described using GFP-tagged PPO2 rather than with the use of antibodies in *ex vivo* hemolymph preparations. The use of the GFP-tagged proteins PPO2 and Tg is shown in additional contexts.

**Table:** Specifications table

Subject area	*Biology*
More specific subject area	*Molecular biology (General), insect immunology, innate immunology*
Type of data	*Images, figures, graphs*
How data was acquired	*Microscopes: LSM 800, Zeiss Axioplan, Leica MZ FLIII fluorescence stereomicroscope*
Data format	*Analyzed* *Filtered* *.docx* *.pdf*
Experimental factors	*Microscopy of GFP fusion constructs from bled Drosophila melanogaster*
Experimental features	*Confocal microscopy, fluorescence microscopy, UAS-GAL4 constructs, RNAi lines, Immunohistochemistry, bleeding, imaging, crossing*
Data source location	*Stockholm, Sweden*
Data accessibility	*Data are supplied with this article*
Related research article	*M. R. Schmid, A. Dziedziech, B. Arefin, T. Kienzle, Z. Wang, M. Akhter, J. Berka, U. Theopold, Insect hemolymph coagulation: kinetics of classically and non-classically secreted clotting factors, Insect Biochemistry and Molecular Biology, 2019.*[1].

**Table:** 

**Value of the data** • The PPO2 fusion construct facilitates the imaging and description of Drosophila crystal cell morphology as well as developmental processes with more accuracy than the PPO antibody. • Crystals appear to be delayed in development and activation and increase in number when glt-RNAi is overexpressed. • Transglutaminase, when expressed with an actin driver, localizes to clot fibers differently from when expressed with a hemocyte driver.

## 1 Data

**Fig. 1:**
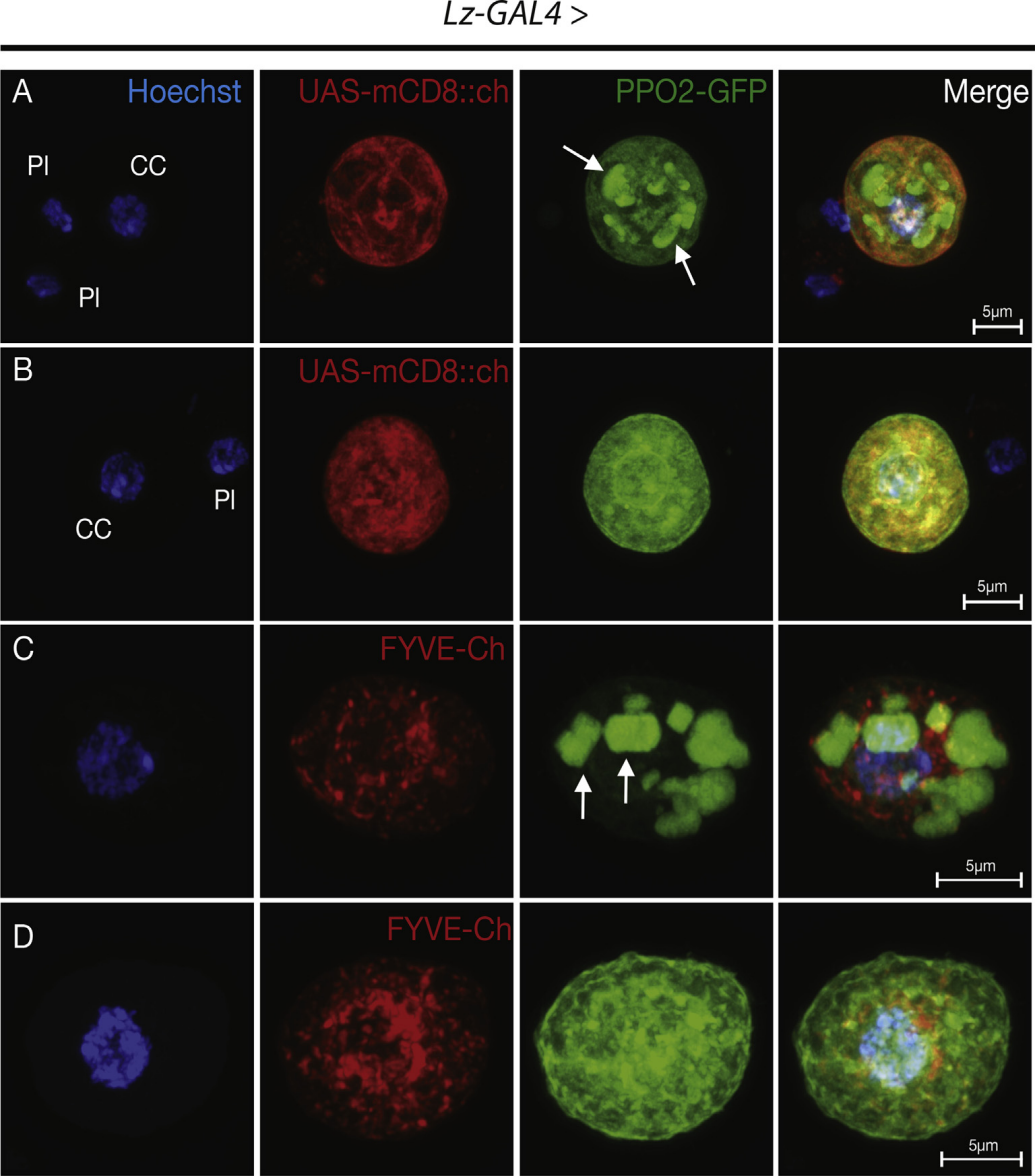
Verification of the PPO2 fusion construct in Crystal Cells (CCs). Using a *Lozenge-Gal4* driver, *PPO2::GFP* (*Lz GAl4 > UAS-PPO2::GFP*) and either *mCD8::Ch* or *FYVE::Ch* were expressed in live CCs and their subcellular localizations were analyzed using epifluorescence microscopy (A–D). PPO2-GFP showed crystals in CCs or different distributions of PPO2-crystals that were defined (arrows in A, C) or mostly cytosolic (B, D). PPO2 did not colocalize with either the membrane marker, mCD8::Ch (A, B) or the FYVE-Ch marker (C, D). The scale bar represents 10 μm. The data show representative images replicated in at least three independent experiments.

**Fig. 2:**
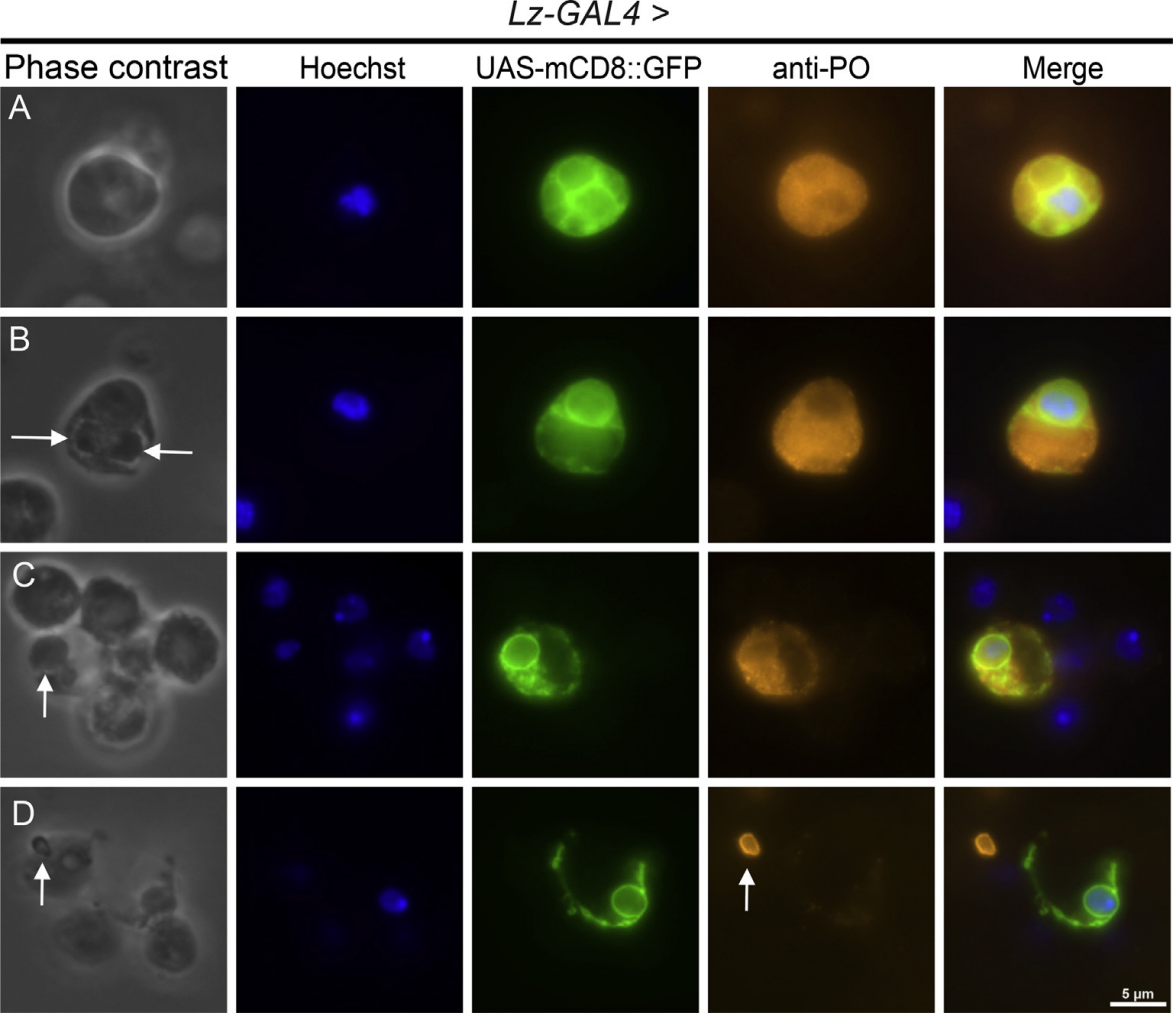
Anti-PPO staining of crystals in fixed Crystal Cells(CCs). *Lz-GAL4 >UAS-mCD8::GFP* was used to identify CCs and crystals were stained overnight with anti-PPO in fixed, permeablized CCs. Different stages of crystal development (arrows in B and C) showed weak staining with the anti-PPO antibody (A–C). Once the membrane integrity of the CC was lost, crystals showed stronger and more specific staining to the antibody (arrow in D). The scale bar represents 5 μm. The data show representative images replicated in at least three independent experiments.

**Fig. 3:**
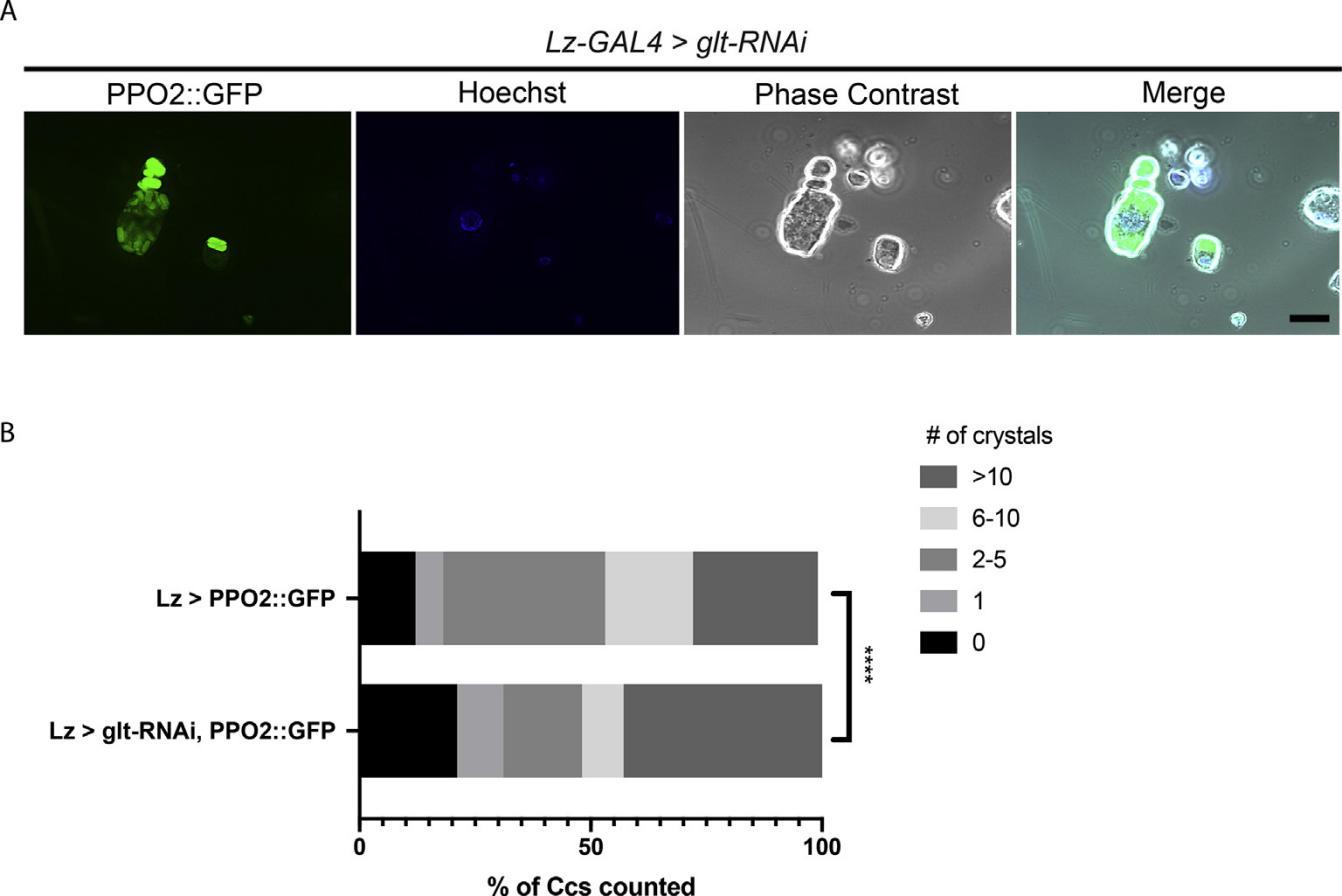
Characterization of the crystal phenotype in Crystal Cells (CCs) expressing *glt-RNAi*. *Lz-Gal4 > glt-RNAi; UAS-PPO2::GFP* larvae were bled, and crystals of CCs were observed to have fractures in the crystals (A). *Lz-Gal4 > PPO2::GFP* larvae and *Lz-Gal4 > glt-RNAi; UAS-PPO2::GFP* were scored for their crystal number distributions and compared. *Lz-Gal4 > glt-RNAi; UAS-PPO2::GFP* larvae had a significantly different distribution of crystals when compared to the control (B). The scale bar represents 10 μm. The data show representative images replicated in at least three independent experiments.

**Fig. 4:**
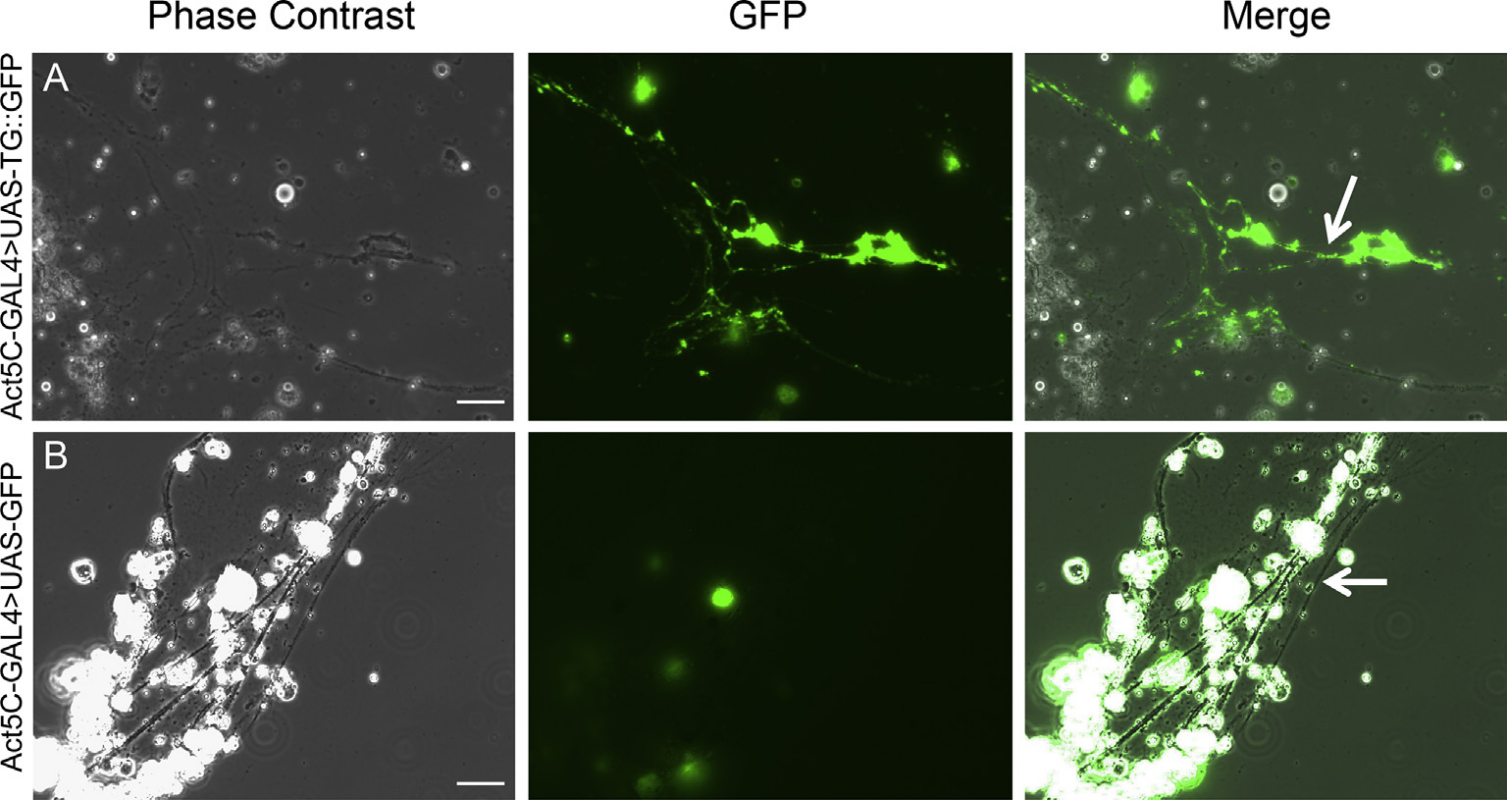
Localization of Transglutaminase in clot fibers using an actin driver. *Actin-Gal4 > UAS-Tg-A::GFP* showed localization to clot fibers (indicated by arrows) compared to *Actin-Gal4 > UAS-::GFP* (compare [1]). The scale bar represents 10 μm. The data show representative images replicated in at least three independent experiments.

The data described here further illuminate the complex features and morphologies of *Drosophila melanogaster* immune blood cells, hemocytes. Two GFP fusion constructs for proteins that are involved in hardening the clot matrix act at different time points, Prophenoloxidase2 (PPO2) and Transglutaminase-A (Tg), were over-expressed using hemocyte specific drivers, *Lz* and *HmlΔ*, as well as a ubiquitous actin driver. The overexpression of *UAS-PPO2*::*GFP* with the *Lozenge* driver in *Drosophila* crystal cells (CCs) was verified in *ex vivo* hemolymph preparations (Fig. 1) and portrayed the shape, number and activation state of the crystals in mature cells (arrows in A and C) and cytosolic expression in immature CCs (Fig. 1B and D). In neither case did membrane markers (mCD8-Ch and FYVE-Ch, [2]) colocalize with PPO2 crystal surfaces. When using a previously described PPO-specific antibody (Fig. 2, [3]), different stages were also detected, although labeling of crystals was more diffuse. In fixed, permeabilized samples, the antibody stained crystals less accurately (Fig. 2A and arrows in Fig. 2B and C) unless the CCs had ruptured exposing the targets for the antibody (Fig. 2D, arrow). The PPO2::GFP construct aided in observing the morphology of crystals when expressing a knockdown of glutactin, a classically secreted basement membrane component (*glt-RNAi*) specifically in crystal cells using the *Lozenge* driver. Crystals, when observed in CCs that were bled into diluted hemolymph, displayed disorganized crystallization (Fig. 3A). The number of crystals per CC were significantly affected by the CC-specific knockdown of classically secreted Glt (Fig. 3B). Finally, the usefulness of these tagged proteins was illustrated when studying how tissue sources other than hemocytes can contribute to the clot. Transglutaminase, when expressed ubiquitously using an actin driver, localized to the clot matrix in contrast to a control, UAS-GFP expression, which remained localized to hemocytes (Fig. 4A and B).

## 2 Experimental design, materials, and methods

### 2.1 Fly strains and husbandry

A standard potato-yeast-agar-sugar diet was used to maintain all *Drosophila* stocks at 25° Celsius. Fly strains, including *w*^*1118iso*^*;UAS-PPO2::GFP*, *w*^*1118iso*^and *UAS-Tg-A::GFP* were created as previously published [1]. The Act5c-Gal4, the mCD8ch, the UAS-EGFP constructs were obtained from Bloomington Fly Center while the *glt-RNAi* line (*w*^*1118*^*; P{GD5017}v15429*) was obtained from the Vienna Fly Center. Experimental crosses were maintained at 25° until third instar wandering larvae had emerged for dissection.

### 2.2 Larval bleeding

Hemolymph was collected from 3 or 5 wandering third instar larvae on a 12-well glass slide containing either 30 or 5 μl of Ringer's solution for either cell imaging or hanging drop preparations, respectively. For cell imaging, cells were allowed to adhere to the glass slide for 10–15 minutes for plasmatocytes or 2–5 minutes for crystal cells and then either fixed with 4% PFA or immediately visualized. For hanging drop/clotting assays, the hemolymph was collected and placed onto 2 mm 36-well slides (Immuno-Cell Int).

### 2.3 Hanging drop and clot collection

After hemolymph was transferred to 36-well slides, the slide was immediately inverted and placed in a humidity chamber, as described previously in [4]. Hanging drops were left for 20 mins to observe melanization or less for clot collection. Clots were collected on 18 × 18 mm coverslips and then placed into 30 μl of Ringer's solution on a glass slide to slow the clotting reaction and to visualize with Hoechst and/or Phalloidin.

### 2.4 Immunohistochemistry

Hemocytes were fixed according to [5] and optimized according to [1]. After adhering to the glass slide, excess Ringer's was removed with a vacuum pump and then fixed with methanol and/or 4% PFA. Cells were then treated with = 0.1% Triton X-100 (Sigma-Aldrich), blocked with 5% Bovine Serum Albumin (BSA, ThermoFischer Scientific) and stained with primary antibodies overnight. The C1 (HC12F6) antibody [3] was used for staining PPO, and an anti-GFP antibody (Invitrogen, 1:500) was used to enhance the GFP signal crystal cell membranes, respectively. The primary antibodies were then visualized with Cy-3 conjugated goat anti-mouse polyclonal antibody or Cy-2 conjugated goat anti-mouse polyclonal antibody (1:600 in 3% BSA, Millipore). Hoechst 33258 (Sigma Aldrich, 1:100) was applied in Ringer's for 5 mins in the final step before adding fluoromount (SouthernBiotech, 100–01) and imaged.

### 2.5 Microscopy

Live hemocytes were imaged with an epifluorescent Axioplan2 light and UV microscope (Carl Zeiss) using a 63× oil immersion objective attached to a Hamamatsu ORCA digital camera operated under Axio Vision Rel.4.8 software. Hanging drops were imaged using a Leica MZ FLIII fluorescence stereomicroscope coupled to a DMC-G2 digital camera (Lumix). Confocal images were visualized with an inverted LSM 800 Airyscan microscope (Zeiss) controlled by ZEN blue 2.1 software and captured with a Plan-Apo 63×/1.40 Oil DIC objective.

### 2.6 Image processing and statistics

Images were processed using either the AxioVision software or the ZEN blue software for epifluorescent and confocal images, respectively. Images were either enhanced in ImageJ version 1.8.0_172 or Adobe Photoshop CS6 version 13.0.6 × 64. Graphs and statistics were produced and assessed using GraphPad Prism software version 8.0. The categorical variables related to the number of crystal cells in *Lz-GAL4>UAS-PPO2::GFP* flies compared to the *Lz-GAL4>glt-RNAi;UAS-PPO2::GFP* flies were assessed using a Chi-Squared test. At a minimum, all experiments were performed in triplicate.
